# Improved protein production and codon optimization analyses in *Escherichia coli* by bicistronic design

**DOI:** 10.1111/1751-7915.13332

**Published:** 2018-11-28

**Authors:** Thijs Nieuwkoop, Nico J. Claassens, John van der Oost

**Affiliations:** ^1^ Laboratory of Microbiology Wageningen University and Research Stippeneng 4 6708 WE Wageningen The Netherlands; ^2^ Max Planck Institute of Molecular Plant Physiology Am Mühlenberg 1 14476 Potsdam‐Golm Germany

## Abstract

Different codon optimization algorithms are available that aim at improving protein production by optimizing translation elongation. In these algorithms, it is generally not considered how the altered protein coding sequence will affect the secondary structure of the corresponding RNA transcript, particularly not the effect on the 5′‐UTR structure and related ribosome binding site availability. This is a serious drawback, because the influence of codon usage on mRNA secondary structures, especially near the start of a gene, may strongly influence translation initiation. In this study, we aim to reduce the effect of codon usage on translation initiation by applying a bicistronic design (BCD) element. Protein production of several codon‐optimized gene variants is tested in parallel for a BCD and a standard monocistronic design (MCD). We demonstrate that these distinct architectures can drastically change the relative performance of different codon optimization algorithms. We conclude that a BCD is indispensable in future studies that aim to reveal the impact of codon optimization and codon usage correlations. Furthermore, irrespective of the algorithm used, using a BCD does improve protein production compared with an MCD. The overall highest expression from BCDs for both GFP and RFP is at least twofold higher than the highest levels found for the MCDs, while for codon variants having very low expression from the MCD, even 10‐fold to 100‐fold increases in expression were achieved by the BCD. This shows the great potential of the BCD element for recombinant protein production.

## Introduction

Heterologous protein production in prokaryotes is one of the major hallmarks of biotechnology and synthetic biology, and it forms the foundation of a wide range of medical and industrial innovations (Elena *et al*., 2014). However, optimization of protein production mostly relies on a trial‐and‐error approach. The poor predictability of high‐level protein production is due to the complexity and interconnection of several determining factors. Key factors at the transcriptional level are the gene's copy number and promoter strength. At the translational level, the ribosome binding site (RBS) strength, mRNA secondary structure and codon usage are key factors that together play a major role in efficient protein production (Kudla *et al*., 2009; Mutalik *et al*., 2013b; Rosano and Ceccarelli, 2014; Quax *et al*., 2015). Especially, factors at the translational level are highly complex, and our limited understanding of these interconnected factors often hampers high protein production (Mutalik *et al*., 2013b; Quax *et al*., 2015).

Translation initiation in prokaryotes occurs when the 16S rRNA of the small ribosomal subunit binds the RBS in the 5'‐UTR of a gene. After this, the large ribosomal subunit is recruited and translation elongation can start. The RBS must be freely accessible to allow recruitment of the ribosomal subunits. Hence, strong secondary structures in the mRNA involving the RBS result in poor ribosome binding kinetics (Studer and Joseph, 2006), which can lead to reduced protein production (de Smit and van Duin, 1990; Kudla *et al*., 2009; Salis *et al*., 2009; Goodman *et al*., 2013). Secondary structures that include the RBS motif have been reported to form either via local contacts between the 5′‐UTR and the adjacent start of the coding domain sequence (CDS), or via long‐range interactions through base pairing of the 5′‐UTR with more distal regions in the CDS (Mustoe *et al*., 2018). A constant 5′‐UTR region can, therefore, perform differently regarding translation efficiency in case of different CDS and 3′‐UTR sequences (Griswold *et al*., 2003). In extreme cases, secondary structures between the RBS and CDS have been reported to block translation completely (Mutalik *et al*., 2013a; Mirzadeh *et al*., 2015).

Given the degeneracy of the genetic code, 61 codons for only 20 amino acids, many different codon sequence variants can encode a certain protein. During translation elongation, codon usage is a crucial factor that can influence the efficiency of protein production in multiple ways. The elongation rate can be limited by several factors such as the availability of cognate aminoacyl‐tRNA's (Hanson and Coller, 2018) and the presence of potential hurdles in the CDS, such as RBS‐like sequences (Li *et al*., 2012; Vasquez *et al*., 2016) and secondary structures (Takyar *et al*., 2005; Buchan and Stansfield, 2007; Chen *et al*., 2013). Coding sequences that are efficiently translated were also reported to be linked to longer mRNA lifetimes, further enhancing production (Boël *et al*., 2016). Whereas in native situations, codon usage has been extensively tuned in the course of evolution, attempts to express such genes at very high‐levels in heterologous production hosts are often hampered. This can potentially be solved by substituting the codons with synonymous counterparts. However, transcript secondary structure and codon sequence are intrinsically correlated. Therefore, the effect of single or multiple synonymous codon substitutions cannot be clearly attributed to changes in translation elongation or in translation initiation (Gustafsson *et al*., 2012; Gorochowski *et al*., 2015).

Many codon optimization algorithms have been developed aiming to improve heterologous protein production (Gould *et al*., 2014), although with varying success rates in terms of increased functional protein production (Maertens *et al*., 2010; Gustafsson *et al*., 2012; Claassens *et al*., 2017; Mignon *et al*., 2018). This variety can be partially explained by the introduction of new secondary structures within the transcript due to synonymous codon changes (Nørholm *et al*., 2013; Mirzadeh *et al*., 2015). Particularly, secondary structures at the 5′‐UTR are overlooked as most optimization algorithms only consider optimization of the CDS and do not take the 5′‐UTR into account. Still, when the 5′‐UTR sequence would be included in the design, currently available tools for RNA secondary structure prediction are not accurate enough to robustly design well‐accessible 5′‐UTRs.

To properly study the effects of codon usage and codon optimization approaches on translation elongation, effects of codons on translation initiation need to be decoupled. To some degree, secondary structures at the 5′‐UTR can be predicted *in silico*, and synonymous codons can be introduced to remove these limitations. However, this requires custom design for each construct and limits codon studies as it dictates codons at the start of the gene. Alternatively, the undesired 5′‐UTR structure may be solved, either on purpose or accidentally, by including well‐expressed N‐terminal protein fusions in the expression vector. These fusions are mostly included to facilitate affinity purification or folding for specific proteins (e.g. His‐tag or MPB‐tag; Griswold *et al*., 2003; Vazquez‐Albacete *et al*., 2017). However, the addition of an N‐terminal peptide to the protein may affect protein functionality and may require additional cleavage and hence is not always a desirable solution.

**Figure 1:**
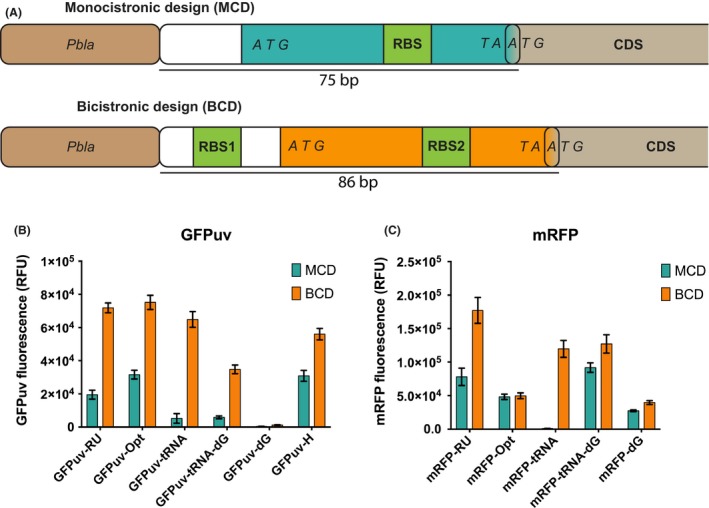
(A) Genetic architecture of monocistronic and bicistronic design. (B,C) The effect of a bicistronic and monocistronic design on the expression of different codon‐optimized GFPuv (B) and mRFP (C) variants (RFU: relative fluorescence units). The regularly used GFPuv (GFPuv‐RU) sequence is compared to an optimized sequence (GFPuv‐Opt), *Escherichia coli* tRNA‐optimized sequence (GFPuv‐tRNA), *E. coli* tRNA‐optimized sequence with subsequent minimalized free energy (GFPuv‐tRNA‐dG), a minimal free energy transcript (GFPuv‐dG) and an *E. coli* harmonized sequence (GFPuv‐H). The regularly used mRFP sequence (mRFP‐RU) is compared with the *E. coli* optimized sequence (mRFP‐Opt), *E. coli* tRNA‐optimized sequence (mRFP‐tRNA), *E. coli* tRNA‐optimized sequence with subsequent minimalized free energy (mRFP‐tRNA‐dG) and a minimal free energy transcript (mRFP‐dG). Production is determined using flow cytometry for eight biological replicates for each variant. The error bars depict the standard deviation for the average expression of eight biological replicates. For each replicate, the expression level of 50 000 single cells is measured, averaged and normalized to a cell culture not expressing any fluorescent protein. For all cases, except mRFP‐Opt, the fluorescence of the BCD variants over the MCD variants is significantly different at a *P*‐value of 0.001. Similar results are obtained for fluorescence measurements obtained with a plate reader (Fig. S4). The MCD and BCD sequence can be found in Table S1.

Therefore, we decided to use a bicistronic design (BCD) element controlling expression of heterologous genes (Makoff and Smallwood, 1990). These elements were previously developed by Mutalik *et al*. (2013a) for reliable generic control of different genes. The BCD contains a well‐accessible RBS1 motif that drives the translation of a short peptide (Fig. 1A). Within the short peptide's CDS, RBS2 is present that allows for translation initiation of the protein of interest, and the stop codon of the peptide sequence overlaps with the start codon of the target CDS. This genetic architecture leads to the translational coupling of the short peptide to the protein of interest (Mutalik *et al*., 2013a). After transcription of the bicistronic mRNA, the ribosome readily binds to the well‐accessible RBS1 site and translates the first cistron; then, the RBS2 site probably becomes available due to the intrinsic helicase activity of the ribosome, irrespective of adverse mRNA secondary structures (Takyar *et al*., 2005).

We here describe the effects of a BCD element on the expression of various codon‐optimized variants of the green fluorescent protein from *Aequorea victoria* jellyfish, optimized for excitation by UV light (GFPuv; Crameri *et al*., 1996), and a monomeric version of the red fluorescent protein from *Discosoma* coral (mRFP; Campbell *et al*., 2002). Both proteins are from eukaryotic origin, which makes them good models for studying codon optimization in a distant bacterial expression host, while their functional expression levels can be easily estimated by measuring fluorescence.

Production from BCDs is compared with production as a single gene (monocistronic design, MCD), the architecture that is generally used for heterologous protein production. We demonstrate that these BCD elements can positively influence the performance of different codon optimization algorithms. Hence, we propose that these BCD elements should be an essential part of future codon usage studies to eliminate the potentially overlapping influence of RNA secondary structure.

## Results and discussion

Various optimized coding sequences for mRFP and GFPuv were expressed using the relatively weak, constitutive beta‐lactamase promoter (*Pbla*). The low transcription rate prevents possible oversaturated gene expression and as such generates a dynamic range that allows for accurately comparing the effects of the used codon optimization strategies and of the BCD and MCD elements. The regularly used (RU) mRFP (Campbell *et al*., 2002) and GFPuv (Crameri *et al*., 1996) sequences, both containing several distinctive mutations compared with the wild type for better stability of fluorescence properties, were compared with several other codon variants, all having identical amino acid sequences to the regularly used protein. These variants include a codon‐harmonized (H) variant (Angov *et al*., 2011), a multiparameter codon‐optimized variant generated using GeneArt's GeneOptimizer software (Opt; Raab *et al*., 2010) and a tRNA codon‐optimized (tRNA) variant (Table S1). Codon harmonization copies the codon usage landscape from the original host to the new host (Angov *et al*., 2011; Claassens *et al*., 2017). GeneArt's GeneOptimizer algorithm performs multiparametric optimization with an apparent preference for common codons as it generated a sequence with the highest Codon Adaptation Index (> 0.9, Table S1; Raab *et al*., 2010). The tRNA codon optimization replaces codons for codons that have the highest number of complimentary tRNA genes. Additionally, a transcript was designed with minimal overall mRNA secondary structure including the fixed 5′UTR and 3′UTR regions (codon usage variants based on this will hereafter be referred to as dG), which allows all possible codons. Lastly, a minimal overall free folding energy transcript was included, which is restricted to codons with well‐represented tRNA's (tRNA‐dG). The harmonized sequence for mRFP is not included, as it could not be designed because the genome of its original host, *Discosoma* sp., is not available.

Protein production overall increases when using a BCD compared with MCD for all GFPuv variants (Fig. 1B). The harmonized and optimized GFPuv sequences resulted in increased protein production compared with the RU sequence in combination with the MCD 5′‐UTR. The tRNA, tRNA‐dG and dG variants with an MCD 5′‐UTR led to lower protein production versus the RU gene variant. However, when comparing protein production of variants expressed with a BCD, completely different relative expression ratios are observed. The harmonized variant performed worse than the RU sequence, and expression of the tRNA‐optimized sequence was similar to that of the RU sequence. The two transcript variants designed to have a low overall free energy (tRNA‐dG and dG) had reduced expression compared with the RU sequence; however, the addition of the BCD improved expression for both variants versus the MCD.

For the mRFP expression similar effects of the BCD were observed. The overall mRFP production improved by the BCD, and relative differences among codon variants are very different compared with the MCD (Fig. 1C). As an exception, the expression of the *E. coli* optimized mRFP did not benefit from the BCD but stayed equal, suggesting that translation initiation is not the limiting factor in this case.

Although the specific codon optimization methods applied in this study were not the main focus, some conclusions can be drawn regarding these methods. First, there is no algorithm that consistently stands out for optimal production of both GFPuv and mRFP. Secondly, a decrease in transcript free energy, especially for the dG variants, seems to lead to reduced expression, possibly due to the incorporation of rare codons in favour of low secondary structures (CAI score < 0.55, Table S1).

**Figure 2:**
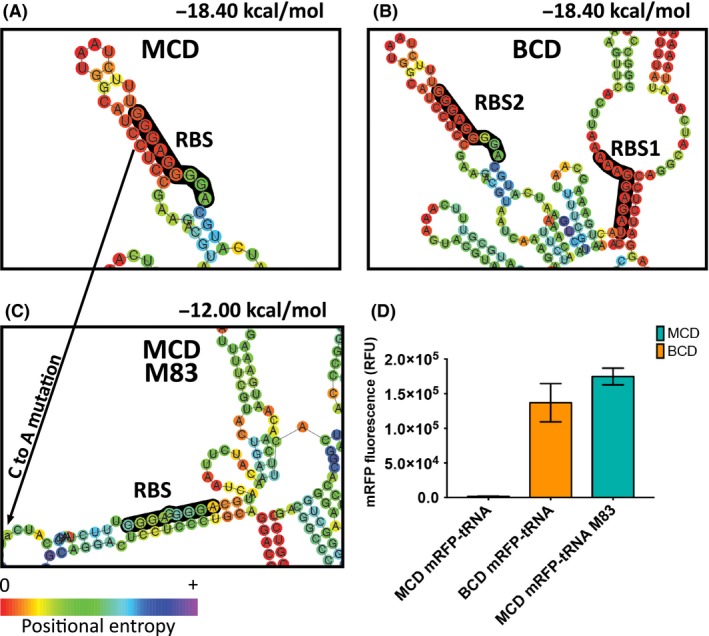
A. Secondary structure prediction of the mRFP‐tRNA transcript with a monocistronic design. The arrow indicates the nucleotide that was silently mutated in an attempt dissolve the structure (M83; C → A. 5′ and 3′ indicate the orientation of the RBS). B. Secondary structure prediction of the mRFP‐tRNA transcript with a bicistronic design. C. Secondary structure prediction of the mRFP‐tRNA M83 transcript with a monocistronic design. The RBS sites are highlighted in black, and the positional entropy for each nucleotide is indicated with a colour gradient. The free energy for each construct is calculated with a sequence window containing the 5′‐UTR and the first 36 nucleotides of the CDS. D. Relative mRFP expression of the mRFP‐tRNA with the MCD, BCD and MCD M83 mutation (RFU: relative fluorescence units). The error bars depict the standard deviation for the average expression of eight biological replicates. For each replicate, the expression level of 50 000 single cells is measured, averaged and normalized to a cell line without mRFP. The mean differences are significantly different at a *P*‐value of 0.001.

In case of the mRFP‐tRNA variant, we further investigated the surprisingly large increase in production from the BCD relative to the MCD (over 100‐fold). The extremely low production from the MCD might be explained by a seriously hampered RBS accessibility. In this specific case, *in silico* secondary structure analysis of the MCD mRFP‐tRNA transcript indeed revealed that the RBS site was involved in a strong loop (Fig. 2A), which could prevent the ribosome from binding. This structure is also predicted in the BCD construct (Fig. 2B); however, the BCD expression appeared not to be affected, as was expected based on the functionality of the BCD architecture that generally prevents issues with RBS2 inaccessibility, probably through the aforementioned ribosome helicase activity (Takyar *et al*., 2005; Mutalik *et al*., 2013a). With an *in silico* prediction; we attempted a design to weaken the RBS‐containing secondary structure by introduction of a silent point mutation in the CDS. (Fig. 2A and C). Experimentally, it could indeed be demonstrated that this mutation indeed recovered expression of mRFP‐tRNA with the MCD, at levels similar to those of the BCD (Fig. 2D).

While the translation initiation limitation for mRFP‐tRNA could be obviously predicted using *in silico* mRNA structure analysis, this was not that obvious for the other expressed GFPuv (Fig. S1) and mRFP (Fig. S2) constructs. Likewise, expression levels for MCD constructs did not correlate with predictions by the RBS Calculator algorithm (Salis *et al*., 2009; Espah Borujeni *et al*., 2014; Fig. S3). This again shows the general limitation of biophysical models and *in silico* tools to design reliable UTR's, whereas the BCD system does not depend on such tools.

Our results show the importance of an accessible RBS region for overall translation efficiency. Due to the intrinsic correlation between the coding sequence and secondary structures of the corresponding mRNA, it will be hard to disentangle these factors in correlation studies. Further, we note that the overall increased expression may also be partly caused by a higher number of ribosomes sequestered to translate the ORF due to the presence of two RBSs. Generally, using a BCD may eliminate the translation initiation as the rate‐limiting step of the translation process. Hence, the BCD approach seems the way to go to study the effect of synonymous codon substitutions on protein production in *E. coli*, and likely also in other prokaryotes. For potential issues related to translation initiation in eukaryotes, different tools will be required, as they rely on fundamentally different translation initiation mechanisms. However, previously developed tools based on upstream open reading frames (uORFP) may be a useful eukaryotic tool (Morris and Geballe, 2000; Ferreira *et al*., 2013), somewhat analogous to BCDs in prokaryotes. Finally, it is concluded that the outcome of the here used codon optimization methods is still rather unpredictable, and better, consistently performing codon optimization algorithms need to be explored, such as by Design of Experiment approaches (Gustafsson *et al*., 2012). An interesting outcome of this study is that the experimental data do confirm the promise of using BCD elements as a generic approach to increase yields in heterologous protein production (Roy *et al*., 2017).

## Conflict of interest

None declared.

## Supporting information


**Fig. S1**. Secondary structure prediction of all GFPuv transcripts.
**Fig. S2**. Secondary structure prediction of all mRFP transcripts.
**Fig. S3**. Translation rate prediction (RBS calculator) versus measured fluorescence.
**Fig. S4**. Correlation between data obtained with flow cytometry and OD_600_ corrected bulk fluorescence using a plate reader.
**Code S1.** Algorithm to reduce the free energy of a transcript using random synonymous mutations.

**Appendix S1.** Describes experimental procedures and materials used in this study.

**Table S1.** Sequence data of constructs used.
**Table S2.** Oligo sequences.
