# Supplementary material for: Improved protein production and codon optimization analyses in Escherichia coli by bicistronic design
Source: Microb Biotechnol. 2018 Nov 28;12(1):173–9. doi: 10.1111/1751-7915.13332 (PMC6302717; doi:10.1111/1751-7915.13332)
Supplement: Supplementary file 1 — Fig. S1. Secondary structure prediction of all GFPuv transcripts. Fig. S2. Secondary structure prediction of all mRFP transcripts. Fig. S3. Translation rate prediction (RBS calculator) versus measured fluorescence. Fig. S4. Correlation between data obtained with flow cytometry and OD600 corrected bulk fluorescence using a plate reader. Code S1. Algorithm to reduce the free energy of a transcript using random synonymous mutations. [file MBT2-12-173-s001.docx]

# Materials and methods

## Strains and media

*E. coli* DH10B (Invitrogen) was used as cloning and expression host. *E. coli* was cultivated in LB (Lysogeny Broth) and LB agar with 50 µg/mL kanamycin when appropriate.

## Strain and plasmid construction

Plasmid pFAB3909(Mutalik *et al.*, 2013)(Addgene #47812) was used as an expression vector. pFAB3909 is designed by Mutalik *et al.* (2013) and contains a bicistronic design (BCD). The BCD5 variant was selected for its relatively high RBS strength and low variance in production for different genes. The promoter sequence was replaced with a bla promoter via PCR using NEB’s Q5^®^ High-Fidelity DNA Polymerase according to the standard protocol. An oligo with the bla promoter as overhang (Oligo 10023, ***Supplementary Table 2***) and phosphorylated oligo (Oligo 10024) bind on either side of the P14 promoter. The resulting amplification fragment is cleaned and concentrated using Zymo’s DNA Clean & Concentrator kit according to the standard protocol. The cleaned-up DNA is ligated using NEB’s T4 DNA Ligase overnight at room temperature. The ligated DNA is transformed into *E. coli* DH10B using heat shock and the sequence is confirmed (Macrogen). The MCD expression vector was constructed by deleting the RBS1 using the same method as described previously (Oligo 10805 and 10806). To introduce the mutation, to abolish the secondary structure around the RBS in pTN0004_mRFP-tRNA, a silent point mutation was introduced using the same method as described previously (Oligo 11665 and 11666). The mRFP(Campbell *et al.*, 2002) and GFPuv sequences were ordered as gBlocks (IDT) and seamlessly cloned into the pFAB3909 vector using NEBuilder® HiFi DNA Assembly Master Mix.

## Protein quantification by flow cytometry

Eight single colonies were picked from an overnight agar plate, containing transformants, and each used to inoculate 200 µl medium in a 2 mL 96 wells plate (Greiner Bio-One, V-bottom). The cultures were grown at 37°C for 18 hours at 200 rpm (1-inch stroke). The cultures were diluted 1000 times in 1x PBS and measured using the Attune NxT flow cytometer (ThermoScientific, software version 2.5). 50,000 single cell events were used to obtain the average mRFP fluorescence (excitation 561 nm, emission 620/15 nm) or GFPuv fluorescence (excitation 405 nm, emission 512/25 nm) for each biological replicate. The mean fluorescence of each replicated was corrected by subtracting the average fluorescence of an *E.coli* strain not expressing mRFP or GFPuv.

## Expression measurement (Plate reader)

Eight colonies were picked from an overnight agar plate, containing transformants, and each used to inoculate 300 µl medium in a 2 mL 96 wells plate (Greiner Bio-One, V-bottom). The cultures were grown at 37°C for 18 hours at 200 rpm (1-inch stroke). 200 µL culture was transferred to a 200 µl 96 wells plate (V-bottom) and centrifugated for 10 minutes at 3800g. The pellets were washed twice with 200 µL 50 mM Tris HCl pH 7.5. 100 µL was transferred to a black sided clear bottom 96 wells plate and the fluorescence was measured using a Synergy Mx plate reader (BIOTEK, software version 3.02.1) (mRFP excitation at 586/9 nm, emission 661/9 nm, gain 125 and GFPuv excitation at 399/9 nm, emission 510/9 nm, gain 75). The fluorescence of each biological replicate was normalized using the OD_600_ and corrected by subtracting the fluorescent value of an *E.coli* strain not expressing mRFP or GFPuv.

## Codon optimization algorithms

**Codon harmonization (H)** of the mRFP and GFPuv CDS has been performed using our online Codon Harmonizer tool (<http://codonharmonizer.systemsbiology.nl>) (Claassens *et al.*, 2017) based on the original algoritm by Angov *et al.*(Angov *et al.*, 2008). **Codon Optimization** has been performed using GeneArt’s GeneOptimizer algorithm web tool (Raab *et al.*, 2010) (performed in May 2017). **tRNA optimization** was performed in-house by replacing all codons with codons that are represented by a tRNA with the highest genome copy number with a preference of Watson-Crick base pairing over wobble base pairing. Genomic tRNA copy numbers for *E. coli* DH10B were derived from <http://gtrnadb.ucsc.edu>. **dG optimization** consisted of random synonymous mutations to lower the overall minimal free folding energy of the construct as much as possible without codon limitations. An in-house script was developed for this purpose (***Supplementary Code 1***). **tRNA-dG optimization** is a combination of the previously mentioned optimization methods. The lowest overall minimal free energy is desired while only codons are selected that are well-represented by tRNA’s.

## mRNA secondary structure analysis

The ViennaRNA Package(Lorenz *et al.*, 2011) was used for all mRNA secondary structure predictions. The RNAfold program (version 2.4.3) was used to generate the minimum free energy secondary structure and base pairing probability matrix. The RNAplot program (version 2.4.3) and Relplot.pl algorithm (version 1.3) were used to draw the RNA secondary structures with base pair probability and highlighted RBS sites.

Angov, E., Hillier, C.J., Kincaid, R.L., and Lyon, J.A. (2008) Heterologous protein expression is enhanced by harmonizing the codon usage frequencies of the target gene with those of the expression host. *PLoS One* **3**: 1–10.

Campbell, R.E., Tour, O., Palmer, A.E., Steinbach, P.A., Baird, G.S., Zacharias, D.A., and Tsien, R.Y. (2002) A monomeric red fluorescent protein. *Proc. Natl. Acad. Sci.* **99**: 7877–7882.

Claassens, N.J., Siliakus, M.F., Spaans, S.K., Creutzburg, S.C.A., Nijsse, B., Schaap, P.J., et al. (2017) Improving heterologous membrane protein production in Escherichia coli by combining transcriptional tuning and codon usage algorithms. *PLoS One* **12**: e0184355.

Lorenz, R., Bernhart, S.H., Höner zu Siederdissen, C., Tafer, H., Flamm, C., Stadler, P.F., and Hofacker, I.L. (2011) ViennaRNA Package 2.0. *Algorithms Mol. Biol.* **6**: 1–14.

Mutalik, V.K., Guimaraes, J.C., Cambray, G., Lam, C., Christoffersen, M.J., Mai, Q.A., et al. (2013) Precise and reliable gene expression via standard transcription and translation initiation elements. *Nat. Methods* **10**: 354–360.

Raab, D., Graf, M., Notka, F., Schödl, T., and Wagner, R. (2010) The GeneOptimizer Algorithm: Using a sliding window approach to cope with the vast sequence space in multiparameter DNA sequence optimization. *Syst. Synth. Biol.* **4**: 215–225.
