# Supplementary material for: Improved protein production and codon optimization analyses in Escherichia coli by bicistronic design
Source: Microb Biotechnol. 2018 Nov 28;12(1):173–9. doi: 10.1111/1751-7915.13332 (PMC6302717; doi:10.1111/1751-7915.13332)
Supplement: Supplementary file 2 — Appendix S1. Describes experimental procedures and materials used in this study. [file MBT2-12-173-s002.docx]

# Supporting information

***
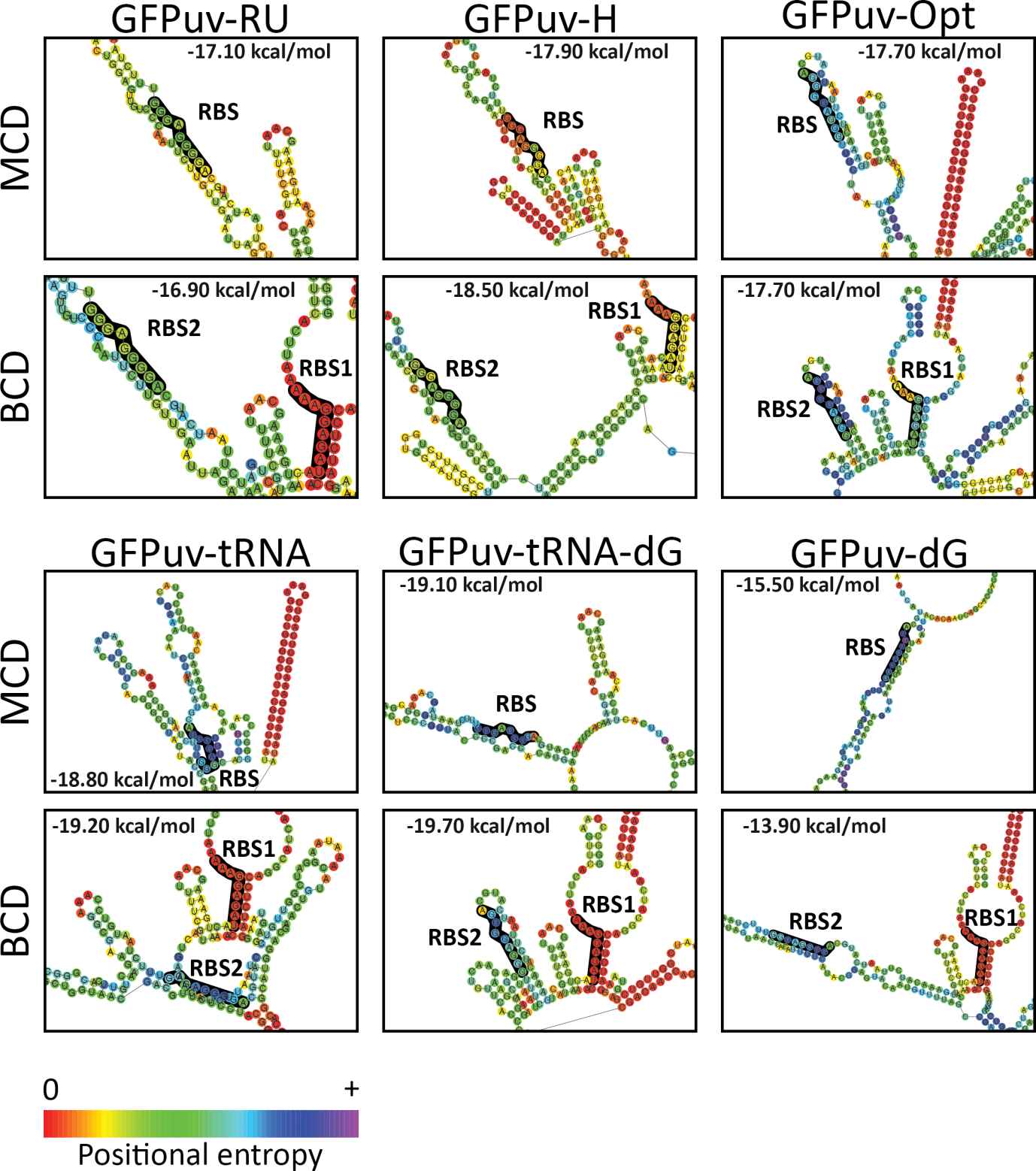
***

***Figure S1*** *5’-UTR secondary structure predictions for differently codon optimized GFPuv transcripts. Either with an MCD element or a BCD element. Ribosome binding sites (RBS) are indicated in black* *and the positional entropy for each nucleotide is indicated with a color gradient. The free energy for each construct is calculated with a sequence window containing the 5’-UTR and the first 36 nucleotides of the CDS.*


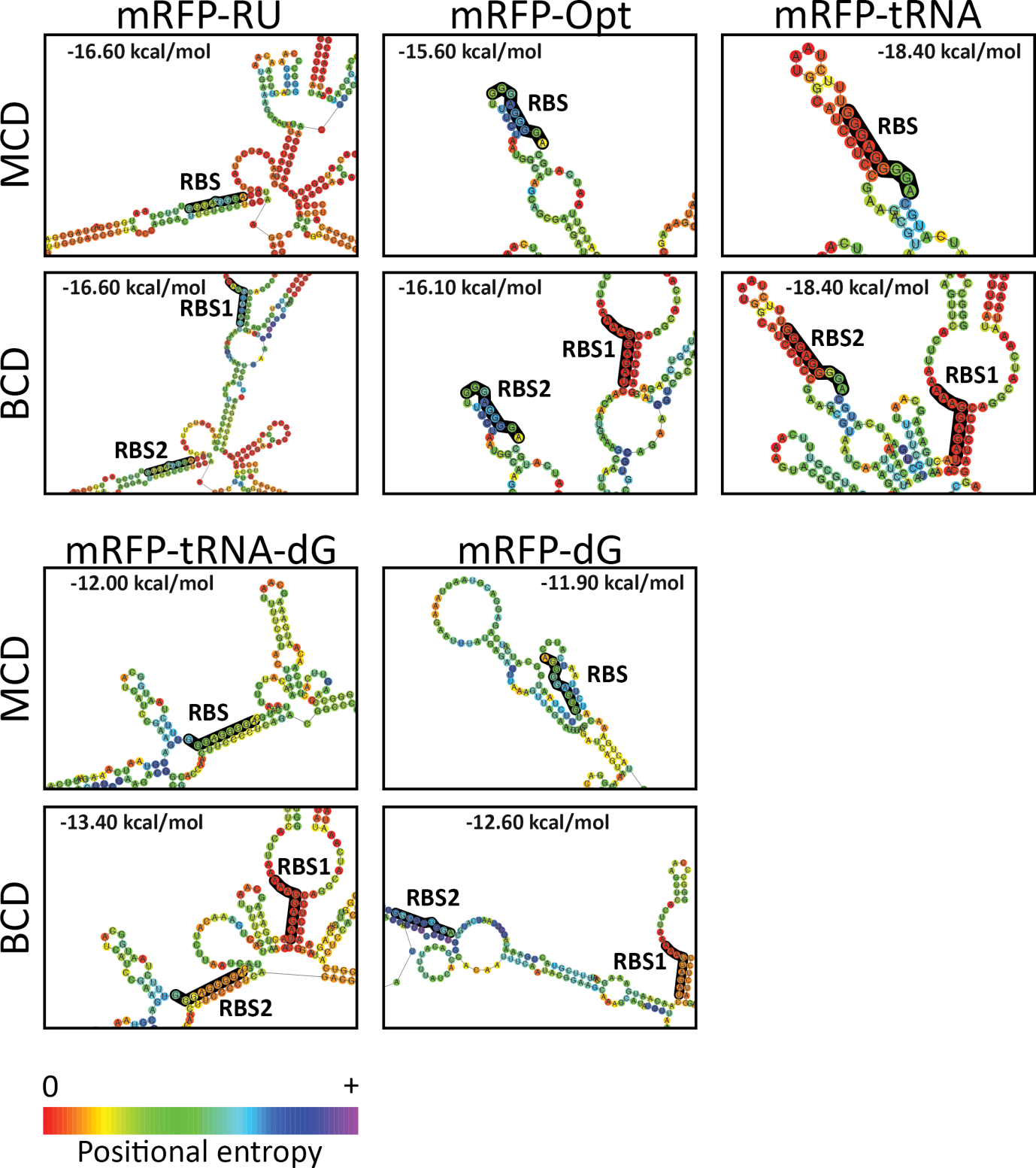


***Figure S2*** *5’-UTR secondary structure predictions for differently codon optimized mRFP transcripts. Either with an MCD element or a BCD element. Ribosome binding sites (RBS) are indicated in black and the positional entropy for each nucleotide is indicated with a color gradient. The free energy for each construct is calculated with a sequence window containing the 5’-UTR and the first 36 nucleotides of the CDS.*

***Figure S3*** *Comparison between predicted translation rate by RBS calculator and experimentally determined expression of protein for MCD constructs. There is no correlation for mRFP (p-value of 0.7275) or GFPuv (p-value of 0.5219).*

***Figure S4*** *Correlation between single-cell data obtained with flow cytometry and OD_600_ corrected bulk fluorescence using a plate reader. Both the measurement for GFPuv (R^2^CV = 0.**824344) and mRFP (R^2^CV = 0.9752014) correlate well.*

***Code S1*** *Algorithm to reduce the free energy of a transcript using random synonymous mutations.*

__author__ = "Thijs Nieuwkoop"

__copyright__ = "Copyright (C) 2018 Thijs Nieuwkoop"

__license__ = "Public Domain"

__version__ = "1.0"

#Writen for Python 2.7.10.

#Requires the ViennaRNA package scripting inferface: https://www.tbi.univie.ac.at/RNA/documentation.html

import sys

sys.path.append("/usr/local/lib/python2.7/site-packages")

import RNA

import random

import time

import datetime

#Settings

name = "GFPuv"

sequence = "ATGAGTAAAGGAGAAGAACTTTTCACTGGAGTTGTCCCAATTCTTGTTGAATTAGATGGTGATGTTAATGGGCACAAATTTTCTGTCAGTGGAGAGGGTGAAGGTGATGCAACATACGGAAAACTTACCCTTAAATTTATTTGCACTACTGGAAAACTACCTGTTCCATGGCCAACACTTGTCACTACTTTCTCTTATGGTGTTCAATGCTTTTCCCGTTATCCGGATCACATGAAACGGCATGACTTTTTCAAGAGTGCCATGCCCGAAGGTTATGTACAGGAACGCACTATATCTTTCAAAGATGACGGGAACTACAAGACGCGTGCTGAAGTCAAGTTTGAAGGTGATACCCTTGTTAATCGTATCGAGTTAAAAGGTATTGATTTTAAAGAAGATGGAAACATTCTCGGACACAAACTGGAGTACAACTATAACTCACACAATGTATACATCACGGCAGACAAACAAAAGAATGGAATCAAAGCTAACTTCAAAATTCGCCACAACATTGAAGATGGATCCGTTCAACTAGCAGACCATTATCAACAAAATACTCCAATTGGCGATGGCCCTGTCCTTTTACCAGACAACCATTACCTGTCGACACAATCTGCCCTTTCGAAAGATCCCAACGAAAAGCGTGACCACATGGTCCTTCTTGAGTTTGTAACTGCTGCTGGGATTACACATGGCATGGATGAGCTCTACAAATAA"

mutation_rate = 5 #mutates between 1 and x codons at the same time.

useSelection = True #True if you want to only use a certain set of codons for replacements.

withFixedUpAndDownstreamRegions = True

upstreamSequence = "GGGCCCAAGTTCACTTAAAAAGGAGATCAACAATGAAAGCAATTTTCGTACTGAAACATCTTAATCATGCAGGGGAGGGTTTCTA"

downstreamSequence = "tgccgactcagttgctgcttctactgggcgccccgcttcggcggggttttttt"

# codons per amino acids in the same order as codonSelection, which are the codons alowed to use for minimalization.

codonInfo = [["gcu","gcc","gca","gcg"],["cgu","cgc","cga","cgg","aga","agg"],["aau","aac"],["gau","gac"],["ugu","ugc"],["caa","cag"],["gaa","gag"],["ggu","ggc","gga","ggg"],["cau","cac"],["auu","auc","aua"],["uua","uug","cuu","cuc","cua","cug"],["aaa","aag"],["aug"],["uuu","uuc"],["ccu","ccc","cca","ccg"],["ucu","ucc","uca","ucg","agu","agc"],["acu","acc","aca","acg"],["ugg"],["uau","uac"],["guu","guc","gua","gug"],["uaa","uga","uag"]]

#For tRNA and min dG (DH10B)

#codonSelection = [["gcc","gca"],["cgu"],["aac"],["gac"],["ugc"],["cag"],["gaa"],["ggc"],["cac"],["auc"],["cug"],["aaa"],["aug"],["uuc"],["ccc","cca","ccg"],["ucc","uca","ucg","agc"],["acc","acg"],["ugg"],["uac"],["gua"],["uaa"]]

#For min dG

codonSelection = [["gcu","gcc","gca","gcg"],["cgu","cgc","cga","cgg","aga","agg"],["aau","aac"],["gau","gac"],["ugu","ugc"],["caa","cag"],["gaa","gag"],["ggu","ggc","gga","ggg"],["cau","cac"],["auu","auc"],["uua","uug","cuu","cuc","cua","cug"],["aaa","aag"],["aug"],["uuu","uuc"],["ccu","ccc","cca","ccg"],["ucu","ucc","uca","ucg","agu","agc"],["acu","acc","aca","acg"],["ugg"],["uau","uac"],["guu","guc","gua","gug"],["uaa"]]

# standardize sequence

upstreamSequence = upstreamSequence.lower()

upstreamSequence = upstreamSequence.replace("t", "u")

downstreamSequence = downstreamSequence.lower()

downstreamSequence = downstreamSequence.replace("t", "u")

sequence = sequence.lower()

sequence = sequence.replace("t", "u")

sequence_correct = True

if len(sequence) % 3 > 0:

print "Sequence length is not a multiple of 3."

sequence_correct = False

for letter in sequence:

if letter not in ['a', 'u', 'c', 'g']:

print "Sequence contains " + letter + " which is not allowed."

sequence_correct = False

if not sequence_correct:

print "Script terminated."

sys.exit()

fixed_sequence = ""

for codon_i in range(0,len(sequence),3):

old_codon = sequence[codon_i:codon_i+3]

found = False

for i, aa in enumerate(codonInfo):

for codon in aa:

if codon == old_codon:

if old_codon in codonSelection[i]:

fixed_sequence += old_codon

else:

print old_codon

fixed_sequence += random.choice(codonSelection[i])

print fixed_sequence

sequence_opt = sequence

sequence_mutation = sequence

if withFixedUpAndDownstreamRegions:

first_data = RNA.fold(upstreamSequence+sequence_opt+downstreamSequence)

else:

first_data = RNA.fold(sequence_opt)

data_string = first_data[0]

free_energy = first_data[1]

def get_new_codon(old_codon):

def find_selection(old_codon):

for i, aa in enumerate(codonInfo):

for codon in aa:

if codon == old_codon:

if useSelection:

return i

else:

return aa

if useSelection:

selection = codonSelection[find_selection(old_codon)][:]

if len(selection) > 1:

if old_codon in selection:

selection.remove(old_codon)

return random.choice(selection)

else:

return random.choice(selection)

else:

selection = find_selection(old_codon)[:]

if len(selection) > 1:

selection.remove(old_codon)

return random.choice(selection)

else:

return old_codon

def mutation_run(sequence_mutation, data_string, free_energy):

mutation_positions = [pos for pos, char in enumerate(data_string[len(upstreamSequence):-len(downstreamSequence)]) if char in ["(", ")"]]

if len(mutation_positions) < 3:

print "Done"

print sequence_mutation

sys.exit()

random_mutation_rate = random.randint(1, mutation_rate)

if random_mutation_rate < len(mutation_positions):

mutation_selection = random.sample(mutation_positions, random_mutation_rate)

else:

mutation_selection = random.sample(mutation_positions, len(mutation_positions))

for mutation in mutation_selection:

mutation_codon_index = mutation - mutation % 3

mutation_codon = sequence_mutation[mutation_codon_index:mutation_codon_index + 3]

new_codon = get_new_codon(mutation_codon)

sequence_mutation_2 = sequence_mutation[:mutation_codon_index] + new_codon + sequence_mutation[mutation_codon_index+3:]

if withFixedUpAndDownstreamRegions:

data = RNA.fold(upstreamSequence+sequence_mutation_2+downstreamSequence)

else:

data = RNA.fold(sequence_mutation_2)

data_string_2 = data[0]

data_free_energy_2 = data[1]

if data_free_energy_2 > free_energy:

print datetime.datetime.fromtimestamp(time.time()).strftime('%Y-%m-%d %H:%M:%S'), name, data_free_energy_2, sequence_mutation_2

return sequence_mutation_2, data_string_2, data_free_energy_2

else:

return sequence_mutation, data_string, free_energy

print datetime.datetime.fromtimestamp(time.time()).strftime('%Y-%m-%d %H:%M:%S'), free_energy, sequence_mutation

print data_string

while True:

sequence_mutation, data_string, free_energy = mutation_run(sequence_mutation, data_string, free_energy)
